# Supplementary material for: Australian general practice registrars perceived development of cultural safety for Indigenous patients: A qualitative study
Source: PLoS One. 2025 Jun 11;20(6):e0325960. doi: 10.1371/journal.pone.0325960 (PMC12157339; doi:10.1371/journal.pone.0325960)
Supplement: Attachment — Submitted filename: PLOS review.pdf [file pone.0325960.s002.pdf]

Feedback on Resubmitted Manuscript

Thank you for the chance to review the updated version of this manuscript. The authors have improved a great deal in response to earlier comments, refining their clarification of the research question, sharpening the discussion, and clarifying the methods section. Nonetheless, some areas remain to be clarified and developed to meet the journal's scholarly standards fully. Following is a list of particular comments in manuscript order.

1. **Introduction**

a) The updated introduction is more concise and has better references but still missing a global context of Indigenous health inequities. The authors can reinforce this by referencing the situations in other countries where the effects of settler colonialism and structural violence continue to impact Indigenous health outcomes.

b) A more explicit sociological perspective may be more usefully applied. The concept of symbolic violence by Pierre Bourdieu may be used to highlight the reproduction of inequalities even in clinical practice that is well-intentioned.

c) The research question is well defined now, yet the section remains to be more specific in stating the gap in the literature. The authors can detail what past research has been limited in doing to make the problem more concise.

2. **Method**

i. Participants and Sampling

a. Although the authors state that 562 registrars were invited and 16 attended, the exclusion and inclusion criteria do not at all appear to be stated clearly. These should be explicitly stated.

b. Also the authors must explain the method of Purposive sampling and the reason behind assuming data saturation with 16 interviews. Connecting this to current theory in qualitative sampling would increase rigor.

ii. Instruments

a. The authors state that the survey and interview measures were assessed for face validity, but this section can be enhanced by stating the methods that were used to design the instruments (e.g., from literature review, theoretical underpinnings, expert consultation).

b. The authors are quiet on the validation of the instrument which raises questions about the data and whether the questions accurately speak to the objective of the study.

c. Although this is a qualitative study, the authors can include a comment on assuring reliability. In qualitative research, these may include iterative testing, triangulation and reflexive questioning refinement all of which enhance trustworthiness.

iii. Interview Modality and Objectivity

a. The rationale behind video-conferencing is sound, though it can be supported further through citing research literature that validates video interviews as a qualitatively acceptable method.

b. Authors should also speak to the limitations of video interviewing that have been documented and detail how these were alleviated.

c. Objectivity and reflexivity receive brief mention. This section may be improved upon by detailing how the research group of authors bracketed their assumptions, whether audit trails and/or collective coding were employed, and what was done in regards to researcher positionality in the interpretative process.

3. **Results**

a. Moving to narrative format is a good decision. It enhances readability.
b. In place of implying participants were “naïve,” the authors could think more critically about what role their limited experience with Indigenous health or training level, cultural context, or international status played in what they reported. These attributes would also account for reasons some topics, like structural racism, did not come up. This part of the text can be informed by literature about how inexperienced clinicians may be reluctant to talk about race, power, or cultural difference either for lack of training or because of the fear of "saying the wrong thing."

4. Discussion

a. The argument is more conceptually solid and evidence-based but authors may also want to comment on how this informs general discussions surrounding decolonisation of health education, most relevant to an international audience.

5. Conclusion

a. The authors made a good attempt with the conclusion but it still ends somewhat broadly. The authors should consider introducing practical steps to be taken as recommendations.
b. Lastly, a brief discussion of how the study adds to international discourse, particularly in other settler-colonial societies will be good for the paper’s global applicability.
