# Supplementary material for: Australian general practice registrars perceived development of cultural safety for Indigenous patients: A qualitative study
Source: PLoS One. 2025 Jun 11;20(6):e0325960. doi: 10.1371/journal.pone.0325960 (PMC12157339; doi:10.1371/journal.pone.0325960)
Supplement: Attachment — Submitted filename: Response to PLOSOne reviewers_final.docx [file pone.0325960.s003.docx]

Responses to reviewers’ comments

| Reviewers comment | Response to reviewer |
| --- | --- |
| 1. Please ensure that your manuscript meets PLOS ONE's style requirements, including those for file naming. | The manuscript has been edited to comply with these requirements. |
| 2. Please include a complete copy of PLOS’ questionnaire on inclusivity in global research in your revised manuscript. Our policy for research in this area aims to improve transparency in the reporting of research performed outside of researchers’ own country or community. The policy applies to researchers who have travelled to a different country to conduct research, research with Indigenous populations or their lands, and research on cultural artefacts. The questionnaire can also be requested at the journal’s discretion for any other submissions, even if these conditions are not met.  Please find more information on the policy and a link to download a blank copy of the questionnaire here: https://journals.plos.org/plosone/s/best-practices-in-research-reporting. Please upload a completed version of your questionnaire as Supporting Information when you resubmit your manuscript. | A completed version of the questionnaire has been submitted as Supporting Information. |
| 3. Thank you for stating the following financial disclosure: This project has received funding through an Australian College of Rural and Remote Medicine Education Research Grant.  Please state what role the funders took in the study.  If the funders had no role, please state: ""The funders had no role in study design, data collection and analysis, decision to publish, or preparation of the manuscript.""  If this statement is not correct you must amend it as needed.  Please include this amended Role of Funder statement in your cover letter; we will change the online submission form on your behalf. | The cover letter has been amended.  The funders had no role in study design, data collection and analysis, decision to publish, or preparation of the manuscript. |
| 4. In the online submission form, you indicated that the datasets are not publicly available due to participants being potentially identifiable from the small dataset but are available from the corresponding author on reasonable request. All PLOS journals now require all data underlying the findings described in their manuscript to be freely available to other researchers, either 1. In a public repository, 2. Within the manuscript itself, or 3. Uploaded as supplementary information. This policy applies to all data except where public deposition would breach compliance with the protocol approved by your research ethics board. If your data cannot be made publicly available for ethical or legal reasons (e.g., public availability would compromise patient privacy), please explain your reasons on resubmission and your exemption request will be escalated for approval. | Freely available data would breach compliance with the protocol approved by the Human Research Ethics Committee.  Participants did not give consent for the data to be publicly available. As the number of Indigenous registrars enrolled with the GP training organisation is very small (approximately five), interview data could be potentially identifiable and attributed to these participants.  Data are available from the research team for researchers who meet the criteria for access to confidential data. |
| 5. Please include captions for your Supporting Information files at the end of your manuscript, and update any in-text citations to match accordingly. Please see our Supporting Information guidelines for more information: http://journals.plos.org/plosone/s/supporting-information. | The manuscript has been edited to comply with these requirements. |
| 1. The introduction must give a global perspective to the discourse on cultural safety before presenting on the regional perspective, herein, Australian Aboriginal and Torres Strait Islander people. | Global perspective added in introduction:  Cultural safety is recognised as a key priority, and driver, for improving anti-racist health care for Indigenous peoples. Indigenous peoples account for just over six percent of the global population and have a life expectancy up to twenty years lower than non-Indigenous peoples (1). As such, improving health outcomes for Indigenous peoples is an international priority. The 2007 United Nations Declaration on the Rights of Indigenous Peoples (2) describes the importance of the right to determine, without discrimination, highest attainable standards of healthcare and access to traditional medicines for Indigenous peoples.  The concept of cultural safety has developed following its introduction by Māori nurse and scholar, Irihapeti Ramsden, in the 1990s. In her thesis, Ramsden asserts cultural safety as:  “*A mechanism which allows the consumer to say whether or not our service is safe for them to approach and use. Safety is a subjective word deliberately chosen to give the power to the consumer. Designed as an educational process by Maori, it is given as a koha [gift or present] to all people who are different from the service providers whether by gender, sexual orientation, economic and educational sta[t]us, age or ethnicity. It is about the analysis of power and not the customs and habits of anybody. In the future it must be the patient who makes the final statement about the quality of care which they receive*” (6) p181.  Since this time, teaching of cultural safety to all health professionals has been recommended in Australia, New Zealand, and Canada (7, 8) but has been complicated, in part, by the wide variety of different terms and definitions in use (9). |
| 2. Present specific objectives or research questions that undergird the study in the last section of the introduction. Ensure that you elucidate why an academic probe into them is timely and relevant. | Specific research question added:  We aimed to explore what factors GP registrars perceived as influencing their development of cultural safety in readiness to consult with Indigenous Australian patients.  Expanded section on why this academic probe is timely and relevant:  Given the lack of evidence about how Australian GP registrars develop, and subsequently apply, cultural safety training to the GP-patient interaction, we considered it an important area to research. Considering the link between cultural safety and improving health of Indigenous Australians, it is important to understand the development of skills in this domain amongst learners who will become a considerable component of the workforce into the future. Appreciating the factors that influence the development of cultural safety may promote targeted training in cultural safety for GP supervisors and medical educators. We particularly wanted to explore this research through the lens of the AHPRA community-derived definition of cultural safety (self-determination, safe, anti-racist, accessible and responsive healthcare, critical reflection of knowledge, skills, attitudes, practicing behaviors and power differentials) (10). |
| 3. In the abstract it was indicated that 16 out of a total of 562 GP registrars were engaged in the study. You must detail this under the methods section, highlighting the inclusion and exclusion criteria for the recruitment of the study participants, the sampling design adopted and what informed the sample size. | Expanded section:  All 562 GP registrars undertaking active training with a Queensland GP training organisation (GPTO), to obtain fellowship with The Royal Australian College of General Practice and/or the Australian College of Rural and Remote Medicine, were invited to participate in this study by email which included links to the participant information sheet, consent form and survey. We purposively sampled one Queensland GP registrar training organisation that agreed to participate in the study after direct approach by the principal investigator. All registrars with this organisation participate in cultural awareness training that is delivered by Indigenous cultural educators. These registrars work within diverse communities, from regional cities to very remote islands, that include approximately 70,000 or two-thirds of Queensland’s Aboriginal and Torres Strait Islander people [48]. Registrars could be training in a range of settings including general practice (mainstream and community-controlled Aboriginal Medical Services), specialty-based hospital disciplines, or rural generalist hospitals. Given the diversity of communities, the high population of Indigenous peoples, and the number of registrars, sampling only one organisation was considered appropriate.  We specified a target sample size of thirty registrars. Following a first round analysis of twelve interviews we decided on a stopping criterion of an additional four interviews that did not yield any new themes of ideas. We considered no new themes were emerging at twelve interviews and as such sixteen registrars participated in this study. |
| 4. There should be a separate section that explains how ethical protocols were duly followed in the conduct of the entire research process including the administration, and designing of instruments as well as in the conduct of the video conferenced interviews. | Ethics section added:  This study was built upon the National Health and Medical Research Council guidelines for the ethical conduct of research with Aboriginal and Torres Strait Islander peoples 47. Support for the project was sought from, and provided by, Aboriginal Community Controlled Health Organisations, the National Cultural Educator and Cultural Mentor Network, and the Leaders in Indigenous Medical Education Network (a national group drawn from the university sector). We consulted with an Indigenous community advisory panel to ensure implementation of culturally sensitive principles throughout the research.  The James Cook University Human Research Ethics Committee approved this study (H8296) following review by Aboriginal and Torres Strait Ethics Advisors in accordance with the National Health and Medical Research Council guidelines.  Participants provided informed consent to completion of both survey and interview. This included name and contact details (email or telephone) to allow the research assistant to contact them to schedule the interview. This identifying information was stored securely and separately to the research data and a unique identifier code used to link the survey and interview data. The research assistant, who contacted the registrars and conducted the interviews, had no prior relationship with the participants but was familiar with their training program. Participants could withdraw from the study at any time with no consequences. Participants were offered a gift voucher to the value of $50 as an acknowledgement of the time they contributed to the study.  A7, an Indigenous researcher, contributed to overall development of this research including development of the survey and interview guide.  Interviews were audio-recorded only. This has been corrected: Audio data was stored securely on a password-protected Microsoft Office One-Drive. Participants consented to data being temporarily stored on Sonix for transcription. |
| 5. The survey instruments (survey and interview guide) must be explained. The scholarly procedures that were followed in the designing of the instruments, the survey questions, and scholarly procedures in validating them before their administration must be explained. | Added:  Registrar demographic details including age, gender, post-graduate level, stage of training, training college, university of graduation, time lived in Australia, exposure to Aboriginal and Torres Strait Islander patients and type of current practice were collected by a Qualtrics® administered survey. This was followed by semi-structured interviews with a research assistant to explore how GP registrars develop cultural safety. The interview guide was developed using the AHPRA definition of cultural safety. The interview guide was reviewed for face validity and appropriateness of content and wording by the whole research team, two independent GPs, and members of the community advisory panel. The survey and interview guide were piloted with one registrar and one GP, and the sequencing of questions were subsequently modified. The research assistant, research team and pilot participants refined the questions and further probes. The research assistant had sufficient experience to modify questions dependent on responses |
| 6. Enough scholarly justifications for the use of video conferenced interviews must be given vis-a-vis it's demerits when compared to face-to-face interviews. More importantly, how was objectivity maintained throughout the conduct of the interviews? These must be discussed. | Added:  Teleconferenced interviews were considered important given the likely geographic distance between research assistant and participants and to allow more equitable access to participation.  We utilized different several approaches to improve the objectivity of our findings, including reflexive journalling, peer debriefing, and member checking. The interviewer discussed their experience, assumptions and perspectives with the research team during fortnightly team meetings. |
| 7. Under the results, authors must interpret the participants' characteristics described and how they may have influenced the data garnered and their analyses. | Added:  Most participants described limited experience within Aboriginal health. This naivete may affect data and analysis. For example, it is possible that this naïveté potentially affected the depth of experiences reported by registrars. This inexperience was not further explored to determine, for example, whether this was under-reporting to adopt a stance of humility/naiveté with the interviewer, a lack of awareness of interaction with Indigenous patients, patients not feeling safe or choosing not to identify, or registrars seeing a skewed patient demographic that participating registrars engaged with. |
| 8. No sound conclusions have been drawn and presented. The authors must draw sound and data-driven conclusions from the key results of the study presented. Also, recommendations for practice could be suggested to GP registrars based on the revelations, though humble, for the essence of cultural safety in medical delivery. | The discussion has been revised to better demonstrate our data-driven conclusions. |
| 9. The manuscript needs to be thoroughly proofread to fix few identified syntax errors. | The manuscript has been proofread and identified syntax errors corrected. |
| As noted in the manuscript, the low response rate resulted in a very small sample size. That said, the importance of this issue, and the insights gleaned from this small sample, render this worthy of publication with the hope that this paper will spark new conversations and, perhaps, follow-up studies with greater participation. | N/A |
| The abstract should be restructured based on the following: Background of the paper should describe the main objective or objectives of the study. You may include the study setting here briefly. method section should briefly explain, which includes research design, study participants, data analysis, etc. Results and findings should summarise the most important results; significance and conclusion should include implications. Refer to the journal guidelines https://journals.plos.org/plosone/s/submission-guidelines  Background/objective: The study does not specify the location or settings where the registrars are practicing (e.g., rural, urban, or remote areas). This context is critical as it influences cultural interactions and healthcare challenges. Design: The following elements are missing, e.g., the authors did not describe how participants were recruited, the sample size, or the inclusion and exclusion criteria. Briefly explain it was used in the study. The explanation of the design may not be necessary in the abstract. Setting: delete and refer to previous comments Participants: delete and refer to previous comment. Results: Briefly state the study findings and implications Conclusion: Include practical public implications. | We have edited the abstract:  There is little evidence of how general practice registrars develop skills towards providing culturally safe care with Indigenous patients. We argue that the patient should determine culturally safe care but not be responsible for teaching or correcting learners. Finding ways to enable this is a challenge for health education systems.  We aimed to explore what factors general practice registrars perceive as influencing their development of cultural safety, in readiness to consult with Indigenous Australian patients. General practice registrars in this study were training in regional, remote and very remote parts of Queensland, Australia, across a vast geographic region.  This qualitative study involved administration of a survey exploring demographic details, alongside semi-structured interviews to explore how general practice registrars develop cultural safety. All general practice registrars with an Australian general practice training organisation were invited to participate in the study. Data collection was conducted from March to August 2022. Transcripts were studied using a thematic analysis approach.  Sixteen registrars completed the survey and semi-structured interview. Registrars described four main factors that contribute towards their development of cultural safety: shared or similar life experiences, learning from cultural safety training, experiential learning, and critical reflection. Registrars thought it was acceptable for patients to educate, teach or correct culturally unsafe care and did not reflect on the power imbalance in these relationships.  Cultural safety appears to be a lifelong learning journey balancing critical self-reflection against an external determination of cultural safety. Understanding this process warrants further research and review of medical education teaching.  Keywords: Cultural safety, Indigenous, general practice, training, registrars, Aboriginal and Torres Strait Islanders |
|  |  |
| Introduction The introduction is succinct and good; however, the authors did not include an example of a health outcome or disparity (e.g., life expectancy, prevalence of specific diseases) to give readers a clearer picture of the issue. Also, the citations are referenced numerically (1, 2), but their specific relevance is not elaborated. It’s unclear where the data comes from or how authoritative it is. | Sentence modified to read:  According to the Australian Institute of Health and Welfare, the effects of colonisation have resulted in this population carrying a disease burden of 2.3 times that of non-Indigenous Australians and a life-expectancy gap of over 8 years when compared to non-Indigenous Australians. |
| Methods Design The authors did not explicitly explain the type of qualitative design used. While it mentions that it is a "qualitative component" of a larger study, it does not specify the exact qualitative approach (e.g., phenomenology, grounded theory, ethnography, case study, narrative analysis). This lack of clarity makes it difficult for readers to understand the framework guiding the research or how data was collected and analysed. | Research design paragraph edited and reads:  We report our qualitative study according to the Consolidated Criteria for Reporting Qualitative Research (16). A detailed description of the methods has been published elsewhere (17). Briefly, this study is part of a larger study exploring assessment of GP registrars’ cultural safety when consulting with Indigenous Australian patients and is shaped by cultural safety (9) and a decolonising approach (18). This qualitative paper reports on four main questions exploring how GP registrars perceive they develop cultural safety to provide appropriate care to Indigenous patients:  How do you feel you developed cultural safety?  What has been the most effective way for you to develop this cultural safety?  What cultural safety (or similar) training have you completed?  What are the main things you have learnt from this training? |
| Participants The number of participants in the study is not explicitly mentioned in the provided text. While 562 GP registrars were invited, it is unclear how many agreed to participate or were included in the final analysis. | The first line of the results reads:  Sixteen registrars participated in this study.  This has been added to the participant section to aid in reading the manuscript. |
| Data collection The authors did not describe how the survey and interview instruments were developed, including whether they were grounded in existing frameworks, literature, or stakeholder input (e.g., Indigenous advisors or GP registrars). There is no mention of how the instruments were tailored to address cultural safety in a manner appropriate for Indigenous contexts. The instruments' reliability and validity are not discussed. Without validation, there is a risk that the tools may not accurately capture the constructs of interest (e.g., cultural safety development).  The authors did not explain how the instruments (survey and interviews) were administered beyond the mode of delivery (e.g., videoconferencing). | Added:  Registrar demographic details including age, gender, post-graduate level, stage of training, training college, university of graduation, time lived in Australia, exposure to Aboriginal and Torres Strait Islander patients and type of current practice were collected by a Qualtrics® administered survey. This was followed by semi-structured interviews, conducted by the research assistant, exploring how GP registrars develop cultural safety. The interview guide was developed using the AHPRA definition of cultural safety. The interview guide was reviewed for face validity and appropriateness of content and wording by the whole research team, two independent GPs, and members of the community advisory panel. The survey and interview guide were piloted with one registrar and one GP, and the sequencing of questions were subsequently modified. The research assistant, research team and pilot participants refined the questions and further probes. The research assistant had sufficient experience to modify questions dependent on responses. Participants were offered face-to-face or teleconferenced interviews with the research assistant. Teleconferenced interviews were considered important given the likely geographic distance between research assistant and participants and to allow more equitable access to participation. Audio data was stored securely on a password protected Microsoft Office One-Drive. Participants consented to data being temporarily stored on Sonix for transcription. The interviewer discussed their experience, assumptions and perspectives with the research team during fortnightly team meetings. |
| Data analysis The authors did not explain how rigour and trustworthiness were ensured during the coding and theme development process. While member checking and team discussions are mentioned, there is no detailed explanation of how biases were minimised, how inter-coder reliability was assessed, or how the "emerging data-driven codes" were systematically validated. | Added:  Rigor and trustworthiness were ensured through regular team meetings amongst the authors to question and agree on the methods, data analysis, conclusions and potential biases. The research assistant and authors engaged in progressive reflexivity checking through discussion/yarning, journalling and peer review. Data was presented at multiple forums, including national and international conference workshops, where conference delegates had opportunity to ask probing questions of the study and analysis, thereby strengthening the findings. |
| Results The combination of tables and plain text in the presentation of the results makes it challenging for readers to follow. I recommend removing the tables and presenting the findings uniformly in plain text to enhance clarity and ensure easier comprehension.  I suggest the authors revise the presentation of the quotes, as they are not accurately or effectively formatted. E.g., I found they were good if they were locally run and they were about my local area that I was working in or being in, but otherwise some of them are not very transferrable. (7400). I also suggest the authors should use pseudonymization instead of numbers. | We have removed the tables and presented the findings in plain text to enhance clarity.  We have revised the quotes to make it easier to read.  We have chosen to avoid the use of pseudonyms recognising that a person’s name can influence a reader’s bias. We have replaced the participant’s unique identifier with Participant A, Participant B etc to avoid using numbers. |
| Discussion The discussion is generally good; however, it lacks implications and suggestions for the phenomenon under investigation. | The discussion has been revised to better demonstrate our data-driven conclusions. |
| Limitations  While the limitation acknowledges the small sample size and geographic focus, it does not explicitly state how these factors affect the generalisability of the findings to other regions, populations, or healthcare settings.  While participation and acquiescence bias are mentioned, potential biases introduced during data collection or analysis (e.g., interviewer influence, coding bias) are not addressed. | This section expanded:  We acknowledge our study has limitations. We recruited only a small sample of GP registrars located in one Australian state which may limit the generalisability of findings. However, these registrars were recruited from a vast geographic region across Queensland. One quarter of the registrars attended medical school outside of Queensland and only half of the registrars had lived in Australia for all their life. This broad range of influences based on geography and past educational experiences suggests that the findings of this study might apply to a broader set of registrars. Our sample may have participation and acquiescence bias (i.e., registrars interested in cultural safety may be more likely to volunteer and give what they perceive as the right answer). If the registrars most interested in cultural safety have volunteered, and they have limited insight about their practice, it is concerning to consider how those who did not volunteer might be practising. In addition, our sample under-represented international medical graduates. Exploring how these registrars view cultural safety is an important area for further research. Further potential biases include researcher reflexivity which we attempted to minimise through regular meetings and peer questioning of our data and interviewer. |
| Conclusion The conclusion lacks specific, actionable recommendations for policymakers, educators, or healthcare organisations on implementing the findings in practice.  The conclusion does not acknowledge the study's limitations or how they might influence the interpretation of findings. | Revised section:  This study highlights the need for ongoing research in this area to further develop ways for patients to determine cultural safety in a GP consultation. Finding ways to enable the patient to determine culturally safe care, but not be responsible for teaching or correcting, is a challenge for health education systems and warrants further review. There is need for further evaluations of cultural safety in GP training considering how cultural safety is both taught and assessed: who determines what is culturally safe care and what are the attributes of the GP registrar, as determined by the Indigenous community, that contribute to a culturally safe practitioner. Culturally safe health care will play an important role in redressing health inequities amongst Indigenous people. Understanding the development of cultural safety amongst learners is an important step towards supporting the development of a culturally safe primary health care workforce. |
